# Editorial: Novel reliable approaches for prediction and clinical decision-making in cancer

**DOI:** 10.3389/fimmu.2024.1537956

**Published:** 2025-01-03

**Authors:** Ines Zidi, Safa Bhar Layeb, Vera Rebmann

**Affiliations:** ^1^ Université de Tunis El Manar, Faculté des Science de Tunis, LR03ES03 Laboratoire des Microorganismes et Biomolécules Active, Tunis, Tunisia; ^2^ LR-OASIS, National Engineering School of Tunis, University of Tunis El Manar, Tunis, Tunisia; ^3^ Centre Génie Industriel, Université Toulouse, IMT Mines Albi, Albi, France; ^4^ Institute for Transfusion Medicine, University Hospital Essen, University of Duisburg-Essen, Essen, Germany

**Keywords:** cancer, systemic analysis, data science, clinical data, prediction, decision-making

Although significant progress has been made in recent decades in understanding the development and progression of cancer, cancer remains one of the leading causes of death. Recent insights on immunobiological dysregulations involved in the development and progression of cancer demonstrate the complexity and heterogeneity of cancer, which play crucial role in the pharmacokinetic variability of cancer therapies. With regard to the prevalence of recurrence/metastases and prognosis, as well as the prediction of cancer treatment success, further investigations are urgently needed to establish cancer signatures or treatment modalities that enable improved risk stratification and improved patient management. This Research Topic focuses on studies that integrate new comprehensive systemic, combinatorial, or complexed data that could be useful to develop personalized treatment regimens, to improve immunotherapies and clinical decision-making.

It is worthy to note that the term ‘complex data’ is usually used to refer to high-dimensional and heterogeneous datasets resulting from several diversified fields involving genomics, transcriptomics, proteomics, and advanced medical imaging techniques such as tomography and contrast-enhanced ultrasound. These data bring important insights into the detailed processes of biological and systemic aspects and are therefore a source of invaluable knowledge for research and clinical applications.

Furthermore, the heterogeneity in data types introduces complexities with respect to data integration and analysis. The interpretation of findings in biologically or clinically relevant contexts then requires the application of sophisticated methods and the use of expert knowledge. This calls for the use of advancing techniques like Artificial intelligence (AI) and machine learning to include deep learning models, which identify patterns and make predictions. Advanced visualization tools go a step further in unraveling such complex relationships.

With the advent of AI, several algorithms are being used to integrate cancer-related data. The study of Wang et al. evaluated multiple machine learning models to build and validate a diagnostic model for patients with advanced adenoma (AA). The XGBoost model identified AA with high sensitivity (70.8%) and specificity (83.4%).

Numerous studies have addressed the pathogenesis of cancer and the phenomena that determine its persistence and progression. In this Research Topic, two studies by Bin Masood et al., and by Zhao and Ren focus on the programmed cell death ligand-1 (PD-L1) marker. Bin Masood et al. demonstrated the importance of plasma PD-L1 testing in the diagnosis of glioblastoma multiforme. Receiver operating characteristic curve analysis was used to calculate the area under the curve for specificity (100%) and sensitivity (57.81%) analysis. Kaplan-Meier survival analysis showed that patients with high PD-L1 levels before surgery had poor overall survival. Zhao and Ren showed that the tracer ^18^F-AlFNOTA-fibroblast activation protein inhibitor (FAPI)-04 in positron emission tomography/computed tomography (PET/CT) SUVsd parameter could predict positive PD-L1 expression in patients with locally advanced esophageal squamous cell carcinoma. Both studies demonstrated the importance of the PD-L1 checkpoint molecule in the targeted therapy by identifying excellent candidates.

Data is also used to analyze the prognosis of cancer patients. The aim of different strategies is to precisely define which patients have a poor prognosis and to be able to easily guide them to other options using a cartesian scientific approach. Wang et al. for example, proposed a prognostic prediction model based on differential gene expression between muscle invasive bladder cancer (BLC) and non-muscle invasive BLC. They reported that the protein S100A9 was significantly elevated in recurrent patients. It may promote BLC cell proliferation, migration, and invasion, and may be a potential therapeutic target for BLC to further support clinical treatment decisions. Li et al. highlighted the importance of the lipid metabolism and immune-related genes in the prognosis of acute myeloid leukemia (AML). They constructed a prognostic signature with hub genes significantly associated with survival using a Gene Set Enrichment Analysis. The created risk signature was negatively correlated with immune cell infiltration. Low-risk patients were more likely to respond to immunotherapy, while high-risk patients responded better to specific targeted drugs. The risk-scoring model is expected to be a valuable tool for individualized treatment decision-making and provide valuable insights to improve patient prognosis and treatment outcomes in AML.

Furthermore, Wang et al. and Liang et al. investigated the value of disulfidptosis, a variant of cell death characterized by disulfide accumulation, in cancer. In fact, Wang et al. computed an optimal predictive model disulfidptosis score (DS) in patients with lung adenocarcinoma. They showed that patients with low DS had a better prognosis, characterized by higher OS, reduced mutation status, improved immune status, and increased sensitivity to immunotherapy. Meanwhile, Liang et al. investigated the predictive value of disulfidptosis-related genes in breast cancer (BC) and their relationship with TME. They constructed a disulfidptosis prognostic model that efficiently predicted BC prognosis.

Four contribution address clinical decision support: The mini-review by Li et al. focuses on hyperprogressive disease (HPD), which occurs in response to immunotherapy with PD-1/PD-L1 immune checkpoint inhibitors (ICIs) in a small proportion of patients with non-small cell lung cancer. It summarizes all aspects of HPD, which is characterized by accelerated tumor growth and early death, including its definition, current biomarkers, potential mechanisms and treatment options. This review provides a detailed insight into the advantages and disadvantages of immunotherapy.

Although immunotherapy, especially PD-1/PD-L1 ICIs, has contributed to a crucial breakthrough in effective cancer treatment, there is still a shortage of surrogate markers or models that guide clinical treatment or predict immunotherapeutic outcomes. For BLC, Xu et al. developed a multidimensional expression regulation model based on immunotherapeutic anti-PD-L1 genes, consisting of the following four genes IGF2BP3, P4HB, RAC3 and CLK2, which predict the efficacy of therapy and identify BLC patients, who will benefit from PD-L1 ICIs therapy. This introduces a new tool for managing BLC.

For advanced hepatocellular carcinoma, Sun et al. established a nomogram in their study that integrates quantitative parameters of tumor characteristics based on pre-treatment contrast-enhanced ultrasound and clinical and laboratory data that predict therapy efficacy of anti-PD-1 in combination with anti-VEGF treatment.

Concerning decision-marking tools for oncologists the study by Xie et al. investigated whether adjuvant therapy (AT) provides an additional benefit for recurrence-free survival (RFS) in patients with squamous cell carcinoma of the esophagus after neoadjuvant chemoimmunotherapy (nICT) and surgery in a multi-center propensity score match study including 155 nICT patients. The results of this study evidenced that postoperative AT is not necessary for an improved RFS in esophageal cancer patients undergoing nICT.

